# SEPPA 3.0—enhanced spatial epitope prediction enabling glycoprotein antigens

**DOI:** 10.1093/nar/gkz413

**Published:** 2019-05-22

**Authors:** Chen Zhou, Zikun Chen, Lu Zhang, Deyu Yan, Tiantian Mao, Kailin Tang, Tianyi Qiu, Zhiwei Cao

**Affiliations:** 1 Shanghai 10th People's Hospital & School of Life Sciences and Technology, Tongji University, Shanghai 200092, China; 2 Shanghai Public Health Clinical Center, Fudan University, Shanghai 200433, China

## Abstract

B-cell epitope information is critical to immune therapy and vaccine design. Protein epitopes can be significantly affected by glycosylation, while no methods have considered this till now. Based on previous versions of Spatial Epitope Prediction of Protein Antigens (SEPPA), we here present an enhanced tool SEPPA 3.0, enabling glycoprotein antigens. Parameters were updated based on the latest and largest dataset. Then, additional micro-environmental features of glycosylation triangles and glycosylation-related amino acid indexes were added as important classifiers, coupled with final calibration based on neighboring antigenicity. Logistic regression model was retained as SEPPA 2.0. The AUC value of 0.794 was obtained through 10-fold cross-validation on internal validation. Independent testing on general protein antigens resulted in AUC of 0.740 with BA (balanced accuracy) of 0.657 as baseline of SEPPA 3.0. Most importantly, when tested on independent glycoprotein antigens only, SEPPA 3.0 gave an AUC of 0.749 and BA of 0.665, leading the top performance among peers. As the first server enabling accurate epitope prediction for glycoproteins, SEPPA 3.0 shows significant advantages over popular peers on both general protein and glycoprotein antigens. It can be accessed at http://bidd2.nus.edu.sg/SEPPA3/ or at http://www.badd-cao.net/seppa3/index.html. Batch query is supported.

## INTRODUCTION

Antigens can be specifically bound by corresponding antibodies through interacting with epitope residues. The identification of B-cell epitopes is of primary importance to vaccine design, immune-diagnostics and antibody production (1). Pioneering work was started by CEP in 2005 (2), then continuous efforts have been paid to epitope prediction in recent years. Currently, several popular tools are available online, such as PEPITO (3), DiscoTope 2.0 (4), Epitopia (5) and SEPPA (6), which were reviewed previously (7). In 2014, SEPPA 2.0 (7) introduced new features of ‘relative ASA preference of unit patch’ and ‘consolidated amino acid index’ to establish a logistic regression model by considering immune host and subcellular localization, demonstrating both increasing accuracy and low false positive rate. Recently, BepiPred 2.0 (8) employed random forest algorithm to predict sequence-based epitopes, achieving an AUC value of 0.596 on external validation set. Besides antigen structures, several methods considered additional information for epitope prediction, such as antibody information (9) or experimental data (10), which is only beneficial to those antigens with prior knowledge. Thus, despite substantial efforts, conformational epitope prediction for protein antigens is still challenging and worthy of further devotion.

In subsequent analysis of 897 immune complexes from PDB database, we found that almost 70% of protein antigens contain N-linked glycosylation sites, indicating the potential to be post-translational modification (PTM) of N-linked glycosylation. It is known that antigenicity of protein antigens, particularly among virus antigens, can be dramatically affected by N-linked glycosylation (11–14). For example, it was reported that key glycan in HIV-1 gp120 surface could block antibody binding and inhibit antibody recognition (15). Also, a group of broadly neutralizing antibodies could directly target the glycan-dependent epitopes located in the V1/V2 region on HIV-1 gp120 (16). In addition, effective tumor vaccines are under development targeting tumor-associated carbohydrate antigens (TACA), which resulted from aberrant glycosylation (17).

It is noted that N-linked glycosylation is the most common forms of protein glycosylation (18), which may influence the local environment physio-chemically, particularly to those neighboring amino acids on protein surface. Yet how to describe the micro-environment featured by glycosylation sites is highly challenging, which has largely hindered the study of epitope prediction involving glycoprotein antigens. So far, no algorithm has considered the effect of glycosylation sites for epitope prediction. Here, we present a latest and enhanced version of SEPPA 3.0, filling the blanks for N-linked glycoproteins. In this version, parameters were refreshed based on the largest dataset till now. Then, the ratio of glycosylation triangles and glycosylation related amino acid indexes were developed and further integrated as new classifiers for glycoprotein antigens. Finally, a calibration parameter was introduced to reduce false positive rate. The performance of SEPPA 3.0 was rigorously evaluated on both glycoprotein and general protein antigens and compared with popular peers.

## DATASET

All datasets were extracted from antigen-antibody complexes in Protein Data Bank (PDB) (19). For quality control, those protein antigens with over 50 residues were remained. Solvent accessible surface areas (SASA) were calculated and epitope residues were defined as the same as SEPPA 2.0 (7). Finally, 832 PDB ID with 897 unique epitope patches were remained. For model construction, 767 protein antigens (520 with N-glycosylation sites) deposited before year 2016 were selected as internal training dataset. Totally, 767 unique epitope structures including 16 544 epitope residues as positive training dataset and 172 975 non-epitope residues as negative training dataset. Two sets were adopted as independent external testing datasets. One refers to general protein antigens without discrimination of N-linked glycosylation or not. This set contains 130 conformational epitopes structures after year 2016, including 2598 positive residues and 31 144 negative ones. Those with N-glycosylation sites were referred as second testing dataset of glycoprotein antigens covering 106 antigens. (Supplementary Table S1).

## MATERIALS AND METHODS

Parameters in SEPPA 2.0 (7) were firstly updated based on the latest training dataset. Two critical parameters, the ratio of glycosylation triangles and glycosylation related amino acid indexes, were added as new classifiers to improve the prediction performance. Then, different machine learning approaches were screened, and logistic regression algorithm was selected to establish our model. Finally, calibration was introduced to reduce false positive rate. The algorithm of SEPPA 3.0 and definition of each parameter are given as below:

### Algorithm of SEPPA 3.0

The functions of sub-model recommendation were remained the same as in SEPPA 2.0 (7). In addition, the prediction algorithm of SEPPA 3.0 was updated as below:

Step 1: determine all the surface residues in the protein antigen;

For each surface residue ${\rm{\ }}r$:

Step 2: search all possible unit triangles within 15 Å atom distance and calculate three triangle-related parameters: propensity index $av{g_r}$ (see SEPPA 1.0), relative ASA $Apre{f_r}$ (see SEPPA 2.0) and ratio of glycosylation triangles $\ Glytr{i_{r\ }}$ using Equation (2);
Step 3: calculate clustering coefficient $\ C{C_r}$ (see SEPPA 1.0), consolidated AAindex value $Inde{x_r}$ (see SEPPA 2.0) and glycosylation related AAindex ($Glyinde{x_r}$) using Equation (3);
Step 4: integrate above six parameters via logistic regression model to present raw antigenicity score for each residue;
Step 5: calibrate raw prediction score using Equation (4) to give the final antigenic score for residue ${\rm{\ }}r$;
Step 6: output the final antigenicity score, and highlight those residues with scores higher than defined threshold. Visualize the subset of predicted epitopes graphically.

### Ratio of glycosylation triangles

The N-glycosylation sites of Asn were defined by sequons of Asn-X-Ser/Thr, where X can be any amino acid apart from proline (20). Surface residue triangle involving Asn of glycosylation sequons was defined as glycosylation triangle ($glytri$). Further, epitope glycosylation triangle ($epi\_glytri$) was defined when it contains at least two epitope residues.

By consolidating 20 amino acids into 13 functional subgroups (21), 71 different glycosylation triangles patterns of subgroups were observed based on training dataset. Ratio of glycosylation triangles ($rati{o_i}$) was intended as its general enrichment in epitope areas as Equation (1) described:

(1) \begin{equation*}rati{o_i} = \frac{{{N_{epi\_glytr{i_i}}}}}{{{N_{glytr{i_i}}}}}\ \left( {i = 1,2, \ldots ,71} \right)\end{equation*}

where $\ {N_{epi\_glytr{i_i}}}$ represents the number of epitope glycosylation triangles pattern ${\rm{\ }}i{\rm{\ }}$ in training dataset, while ${N_{glytr{i_i}}}$ is the number of glycosylation triangles pattern ${\rm{\ }}i$ in training dataset.

For each surface residue $\ r$, search all possible ${\rm{\ }}glytri$ within 15 Å atom distance and define the occurrences times of specific glycosylation triangle pattern $\ i$ as $\ {N_{occur( i )}}$. The weighted score for glycosylation triangle pattern $i$ ($glytr{i_i}$) was defined as $\ \ {W_{ri}} = \ rati{o_i}\ *{N_{occur( i )}}$ and scores of 71 existing ${\rm{\ }}glytri$ pattern were integrated by artificial neural networks (ANNs) into a consolidated ratio of glycosylation triangles as Equation (2) described:

(2) \begin{eqnarray*}Glytr{i_r} &=& NN \lbrace{{W_{r1}},\ {W_{r2}}, \ldots ,{W_{ri}}, \ldots ,{W_{r71}}}\rbrace \nonumber\\ &&\,({i\ = \ 1,2, \ldots ,71})\end{eqnarray*}

where $\ {W_{ri}}$ means the weighted score of $glytri$ pattern ${\rm{\ }}i$, while $Glytr{i_{r\ }}$ indicates the consolidated ratio of glycosylation triangles via two-layer and ten-node ANN.

### Glycosylation related amino acid indexes

Statistical analysis was done to derive four glycosylation-related amino acid indexes from AAindex database (22) as initial features classifying epitope residues from non-epitope surface ones, including KIMC930101, LAWE840101, RICJ880102 and ROBB760113 (Supplementary Figure S1). Then, shell structure model was generated to summarize different amino acid indexes into each layer (23). Based on the radius of 10 Å and step size of 2 Å, five layers were generated for each residue ${\rm{\ }}r$. For each index ${\rm{\ }}i$ of residue ${\rm{\ }}r$, the amino acid index of all residues within layer ${\rm{\ }}j{\rm{\ }}$ were averaged to generate a glycosylation-related index as ${\rm{\ }}glyinde{x_{ij}}$. Finally, 20 indexes (4 indexes * 5 layers) were optimized through iterative filtering, and the optimized combination of glycosylation-related amino acid indexes were consolidated by ANNs as Equation (3) illustrated:

(3) \begin{eqnarray*}Glyinde{x_r} &=& NN \lbrace{{optimize}}\nonumber\\ &&\,{{( {glyinde{x_{11}}, \ldots ,glyinde{x_{ij}}, \ldots ,glyinde{x_{45}}})}}\rbrace \end{eqnarray*}

where ${\rm{\ }}i$ represents four types of AAindexes and $j$ represents five layers of shell model, $glyinde{x_{ij}}$ means the averaged index ${\rm{\ }}i$ within layer ${\rm{\ }}j$ of shell model, and $Glyinde{x_r}$ indicates glycosylation-related AAindexes of residue ${\rm{\ }}r$.

### Calibration parameter

The idea of calibration is to adjust the raw score of individual residues by the overall tendency of neighboring residues, such as a low-score/high-score residue sitting in high-epitope/low-epitope environment. The adjusted score of residue ${\rm{\ }}r$ was defined by the average score of all neighboring surface residues as Equation (4) illustrated:

(4) \begin{equation*}adjust\_scor{e_r} = \frac{\sum raw\_ scor{e_i}}{N}\end{equation*}

where ${\rm{\ }}\sum raw\_scor{e_i}$ represents the sum of raw predicted scores for each neighboring surface residue within 5 Å atom distance of target residue ${\rm{\ }}r$, while ${\rm{\ }}N$ means the total number of above residues.

## RESULTS

### Internal validation of SEPPA 3.0

The performance of SEPPA 3.0 was rigorously validated through both internal and external validation. As being illustrated in SEPPA 2.0 (7), area under curve (AUC) value and balanced accuracy (BA) were adopted as evaluation parameters. For internal validation, five commonly used machine learning approaches were screened, including logistic regression, naïve Bayes, random forest, support vector machine and decision tree. The results of 10-fold cross-validation showed that logistic regression gave the best prediction results with AUC value over 0.79 for general protein antigens (Supplementary Figure S2), and was chosen for model construction of SEPPA 3.0.

### Comparing with peer methods

Further, SEPPA 3.0 was evaluated through independent testing dataset and further compared with six available peers on-line with two sets of independent testing data. Peers include Epitopia (5), Discotope-2.0 (4), Pepito (3), CBTOPE (24), SEPPA 2.0 (7) and BepiPred-2.0 (8). Two independent testing sets include: (i) group 1 contains 130 general protein antigens (G1) and (ii) group 2 contains 106 glycoprotein antigens (G2). Note that there is no recommended threshold from Epitopia (5), the optimized threshold with maximum BA on different testing set was purposely selected for Epitopia (5). The results of evaluation on G1 general protein antigens were illustrated in Supplementary Figure S3. Pepito (3) achieved the best performance among six previous peers with AUC value of 0.660, while SEPPA 3.0 gave final AUC of 0.740 on general protein antigens. By setting the default threshold, SEPPA 3.0 achieved the highest BA of 0.657 among all others.

**Figure 1.**
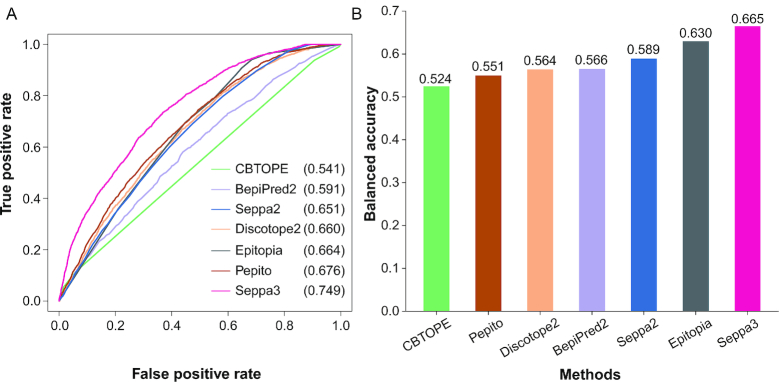
Performance comparison between SEPPA 3.0 and available peers. (**A**) ROC curves on independent testing of 106 glycoprotein antigens. (**B**) Balanced accuracy on independent testing of 106 glycoprotein antigens.

Further performance was evaluated on G2 glycoprotein antigens (Figure 1). The AUC was pushed up to 0.749 by SEPPA 3.0, leading the most accurate prediction for glycoprotein antigens, while the second winner is Pepito (3) with AUC of 0.676. Thus, SEPPA 3.0 shows significant advantages over popular peers on both general protein and glycoprotein antigens. Detailed performance of SEPPA 3.0 and available peers can be found in Supplementary Table S2.

### Case study

**Figure 2.**
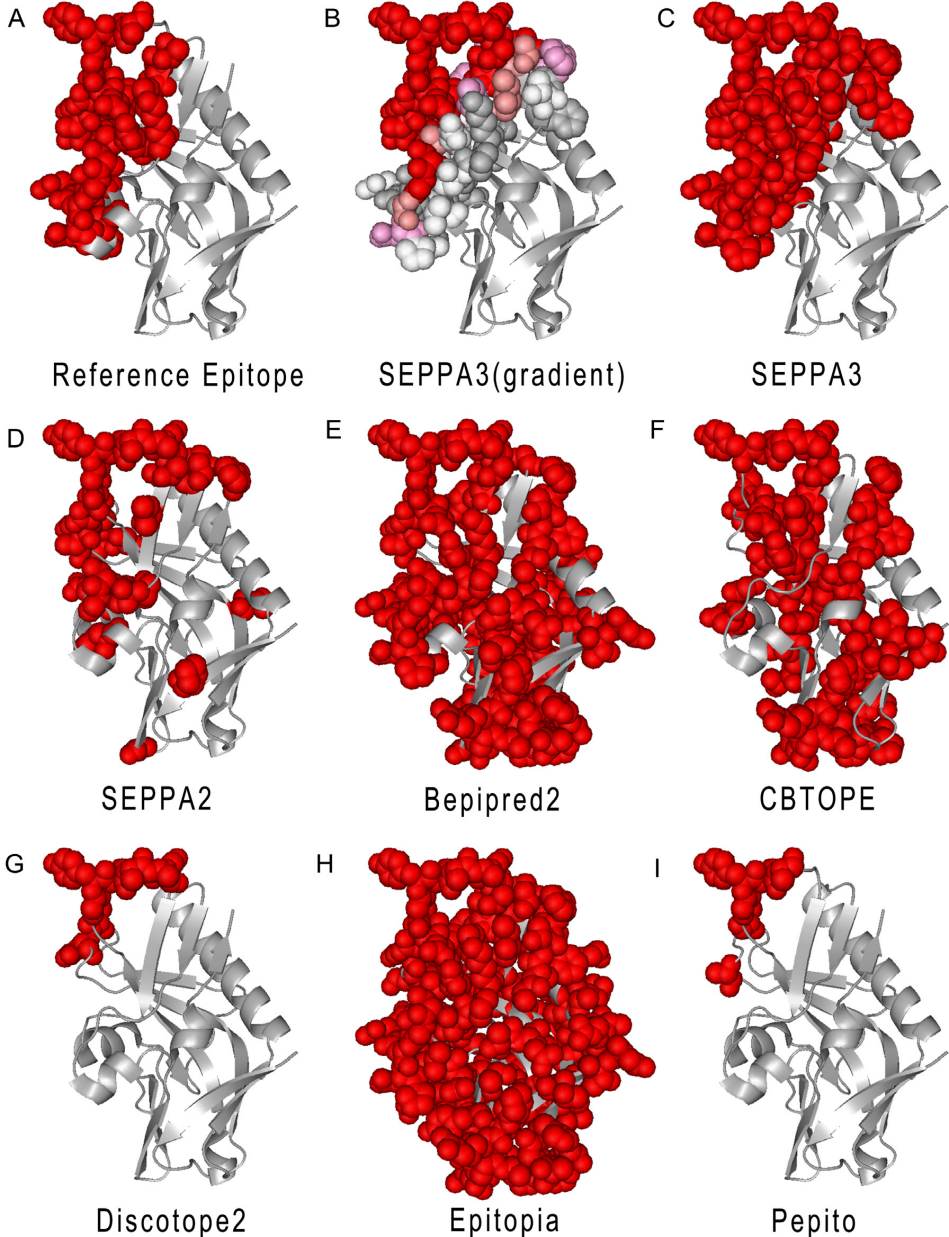
Case study of HIV-1 gp120. (**A**) Reference epitopes of gp120 (PDB ID: 5IF0, Chain: G). (**B**) Predicted epitope residues for HIV-1 gp120 by SEPPA 3.0 with gradient illustration under default cutoff. Red, salmon, pink, white and grey color illustrate those candidates from high score to low score. (**C**) Predicted epitope residues for HIV-1 gp120 by SEPPA 3.0 with unified color under default cutoff. (**D**) Predicted epitope residues for HIV-1 gp120 by SEPPA 2.0. (**E**) Predicted epitope residues for HIV-1 gp120 by Bepipred-2.0. (**F**) Predicted epitope residues for HIV-1 gp120 by CBTOPE. (**G**) Predicted epitope residues for HIV-1 gp120 by Discotope-2.0. (**H**) Predicted epitope residues for HIV-1 gp120 by Epitopia. (**I**) Predicted epitope residues for HIV-1 gp120 by Pepito.

Here, the well-known gp120 glycoprotein antigen (PDB ID: 5IF0, Chain: G) was selected as input and the results were illustrated in Figure 2. Gp120 contains 30 epitope residues according to the complex structures from PDB (Figure 2a), out of which, 29 were predicted as true positive by SEPPA 3.0. Under the default threshold of 0.089, though 60 residues were calculated as potential epitope residues, most of true epitope residues were ranked in the top list (Figure 2B). For example, by raising the threshold from 0.089 to 0.29, top 16 ranking marked in red contains 15 true epitope residues, indicating the excellent performance of SEPPA 3.0. The prediction results of 5IF0_G could reach to an AUC of 0.936 with BA of 0.872. Also, the predicted epitopes of SEPPA 2.0 and other peers including Bepipred-2.0, CBTOPE, Discotope-2.0, Epitopia and Pepito were illustrated in Figure 2D to I, under default cut-offs respectively. Results illustrated that, compared with other peers including SEPPA 2.0, SEPPA 3.0 gave the best results which are most similar to reference epitopes. Meanwhile, those false positive of discrete candidates predicted by SEPPA 2.0 can be successfully adjusted through the algorithm of SEPPA 3.0. Besides the case of HIV-1 gp120, another example was also illustrated (Supplementary Figure S4) based on human glycoprotein antigens of CD27 (PDB ID: 5TLK Chain: X), which is an important antibody drug targets for autoimmune diseases and cancers (25). Model performance of 130 individual structures from testing dataset can be found in Supplementary Tables S3 and S4.

## USAGE

### Input

SEPPA 3.0 (http://bidd2.nus.edu.sg/SEPPA3/ or http://www.badd-cao.net/seppa3/index.html) accepts two types of input files: (i) Existing PDB IDs with chain name, and (ii) Local files in PDB format. Like SEPPA 2.0 (7), users are recommended to select subcellular localization of protein antigen and species of immune host if available. Also, batch query submission is encouraged. Users can submit multiple PDB IDs in batch query with specified PDB IDs, subcellular localization, species of immune host and chain name as input. After successful submission, SEPPA 3.0 will automatically recommend the best model based on user's specifications.

### Output

**Figure 3.**
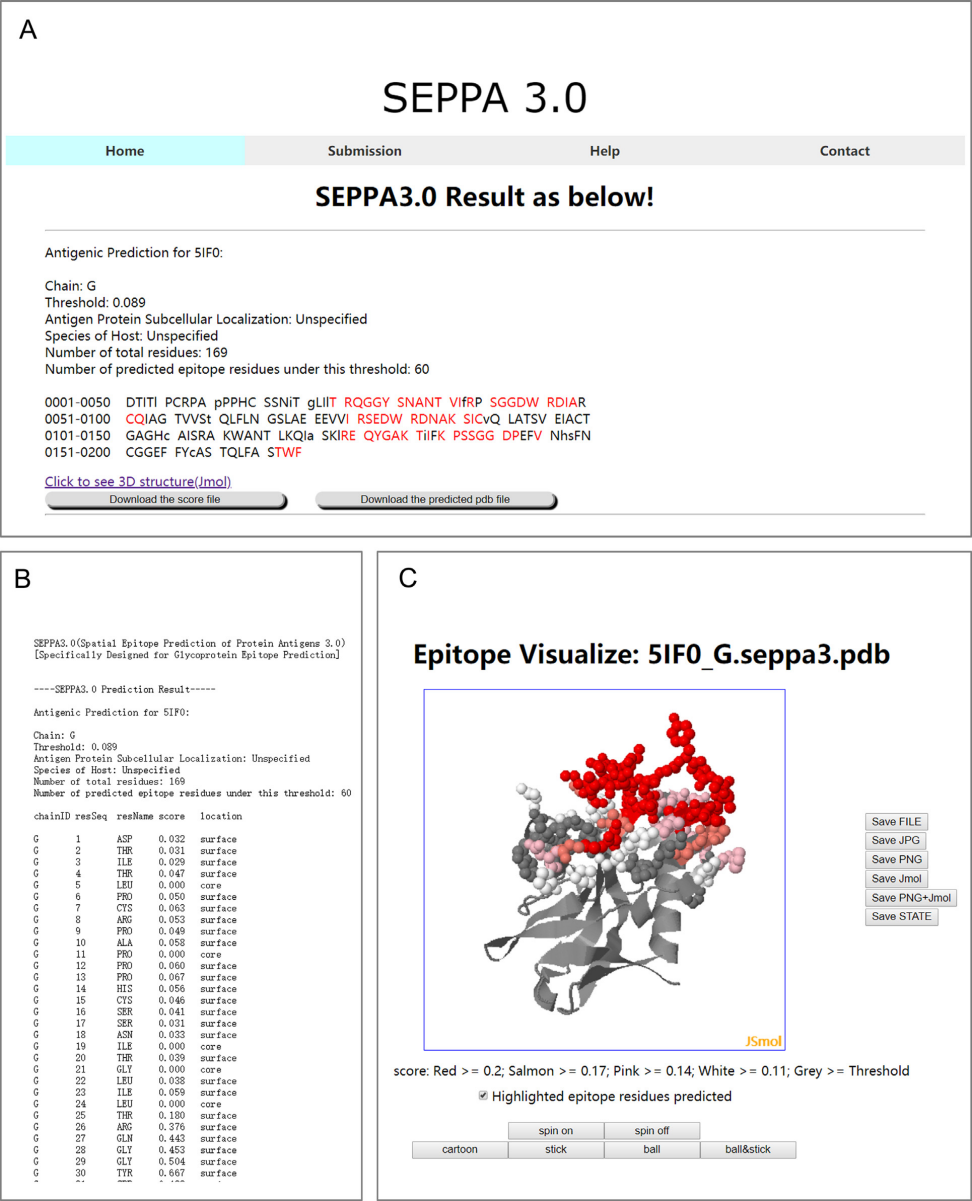
Illustration of SEPPA 3.0 output results. (**A**) Result summary for epitope prediction of query antigen. (**B**) Antigenicity scores predicted for each residue in query antigen. (**C**) 3D visualization of query antigen.

The output results of SEPPA 3.0 will be either presented in .html format or be sent back to users via email (Figure 3). The .html format will provide result summary of input files in sequence level, in which capital letter and lowercase represent the surface and core residues respectively. Possible epitope residues predicted were marked in red capital letters (Figure 3A) and detailed antigenicity scores calculated were recorded in score file for each residue in query antigen (Figure 3B). Users can visualize the prediction results through online plug-in Jsmol components or local PDB files downloadable. Here, HIV-1 gp120 (PDB ID: 5IF0, Chain: G) is shown as an example to illustrate the predicted results of SEPPA 3.0. Residues were colored according to predicted scores (Figure 3C). More information can be found in the HELP page of SEPPA 3.0.

## DISCUSSION

In recent decade, the progress in conformational epitope prediction goes smoothly but slowly. The space to improve may lie in several aspects, including accumulation of more epitope structures, appropriate classifying features, more refined models for specific datasets, and so on. In order to maintain the top performance, SEPPA 3.0 updated the largest datasets, the latest features, and designed two new classifiers for N-linked glycoprotein antigens. Our statistical analysis showed that N-glycosylation sites were significantly enriched in epitope areas rather than in non-epitope surface regions (paired *t* test, *P* = 3.577e–14, 95% CI = [0.032, 0.051]), suggesting that antibodies seem to prefer certain surface region containing N-glycosylation sites. In other words, comparing to those in non-epitope surface areas, N-glycosylation sites in epitope regions might have different local context residues layout, as well as physio-chemical fields resulted from neighboring residues. Thus two types of parameters were designed to test the potential difference of the micro-environment nearing glycosylation sites between epitope areas and non-epitope surface regions. After statistical analysis, the unit patch of residue triangles (6) involving N-glycosylation sites and four amino acid indexes were selected out to indicate residues layout and physio-chemical fields respectively. Meanwhile, by introducing shell structure to construct the glycosylation-related AAindexes, the layers of micro-environmental variations were fully considered and summarized for each residue. Despite of the diverse features invented till now, predicting results can only be lifted by combining multiple of them, suggesting the inherent complexity of epitope nature.

Another task for epitope prediction is to reduce the high False Positive Rate (FPR). Most available algorithms calculated epitope scores based on each individual residue. As structural epitope areas are surface patches being recognized and bound by CDR regions of antibodies, collective effects might play much more important roles than we had expected. Differed from other popular peers, SEPPA 3.0 designed the parameters of the local structural layout and micro-environment around each target residue to describe the collective effects in epitope regions. More importantly, a calibration procedure was elaborated to further reduce FPR based on neighboring influence. In fact, after calibration, the FPR of SEPPA 3.0 was significantly decreased from 0.35 to 0.26 for general protein antigens and from 0.34 to 0.25 for glycoprotein antigens.

For binary classification problems, AUC value was often adopted to evaluate the model performance under different thresholds. Another evaluation parameter we took is balanced accuracy, which fully considered the balance between sensitivity and specificity. Using the above two parameters, the performance of different tools can be fairly compared on independent datasets. It is noted that the structure information of conformational epitope is rapidly accumulating. Coupled with new classifiers and FPR reducing methods, it is expected that epitope prediction models could better serve the biological needs and assist the process of potential vaccine and immune therapeutic development.

## DATA AVAILABILITY

PDB ID: 5IF0, 5TLK.

## Supplementary Material

gkz413_Supplemental_Files
