# Supplementary material for: SEPPA 3.0—enhanced spatial epitope prediction enabling glycoprotein antigens
Source: Nucleic Acids Res. 2019 May 22;47(W1):W388–94. doi: 10.1093/nar/gkz413 (PMC6602482; doi:10.1093/nar/gkz413)
Supplement: gkz413_Supplemental_Files [file gkz413_supplemental_files.pdf]

Supplementary Figures

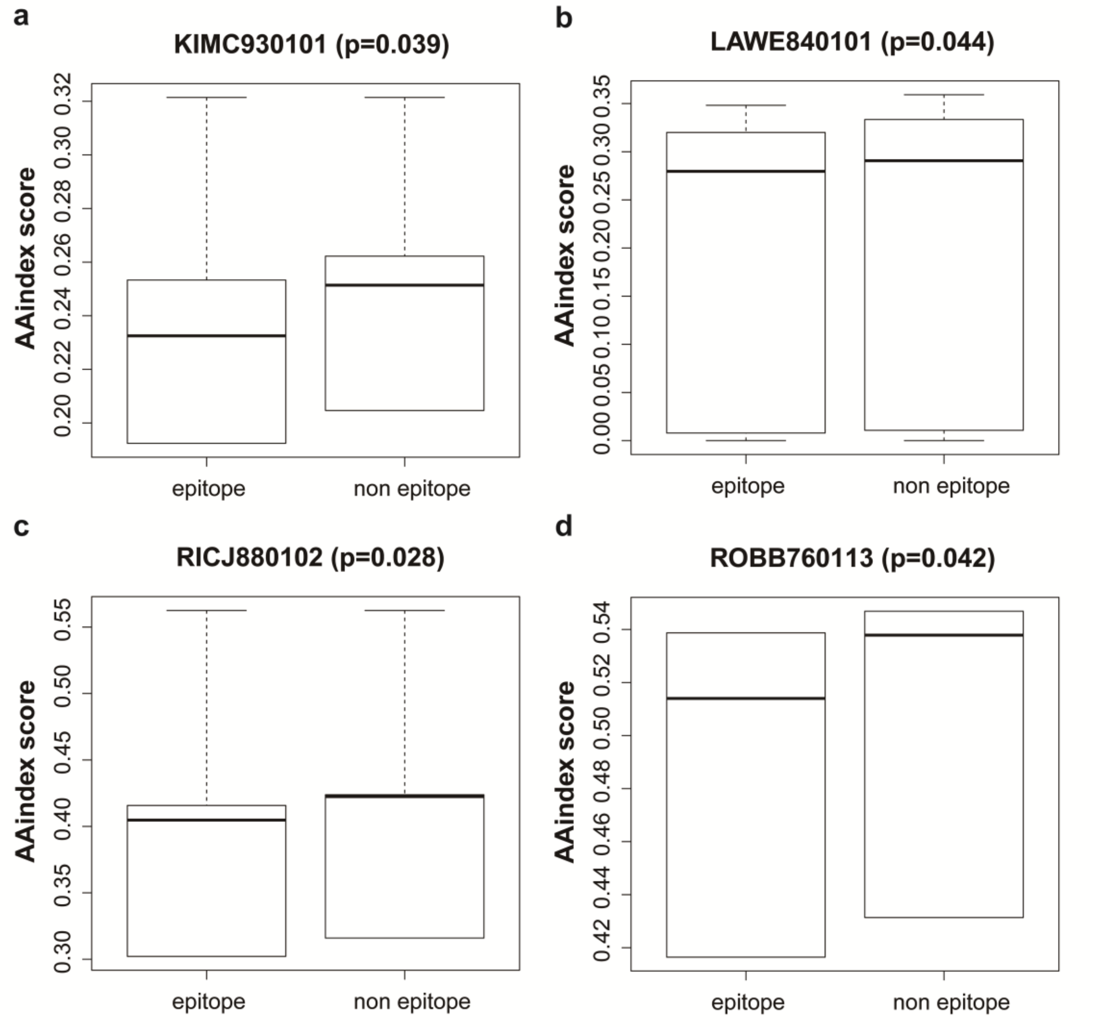

Figure S1. Statistical test of physic-chemical properties between epitope and non-epitope regions (paired t test). (a) Box plot of AAindex KIMC930101 (95% CI = [-0.025, -0.001]). (b) Box plot of AAindex LAWE840101 (95% CI = [-0.015, -0.001]). (c) Box plot of RICJ880102 (95% CI = [-0.021, -0.002]). (d) Box plot of AAindex ROBB760113 (95% CI = [-0.022, -0.001]).

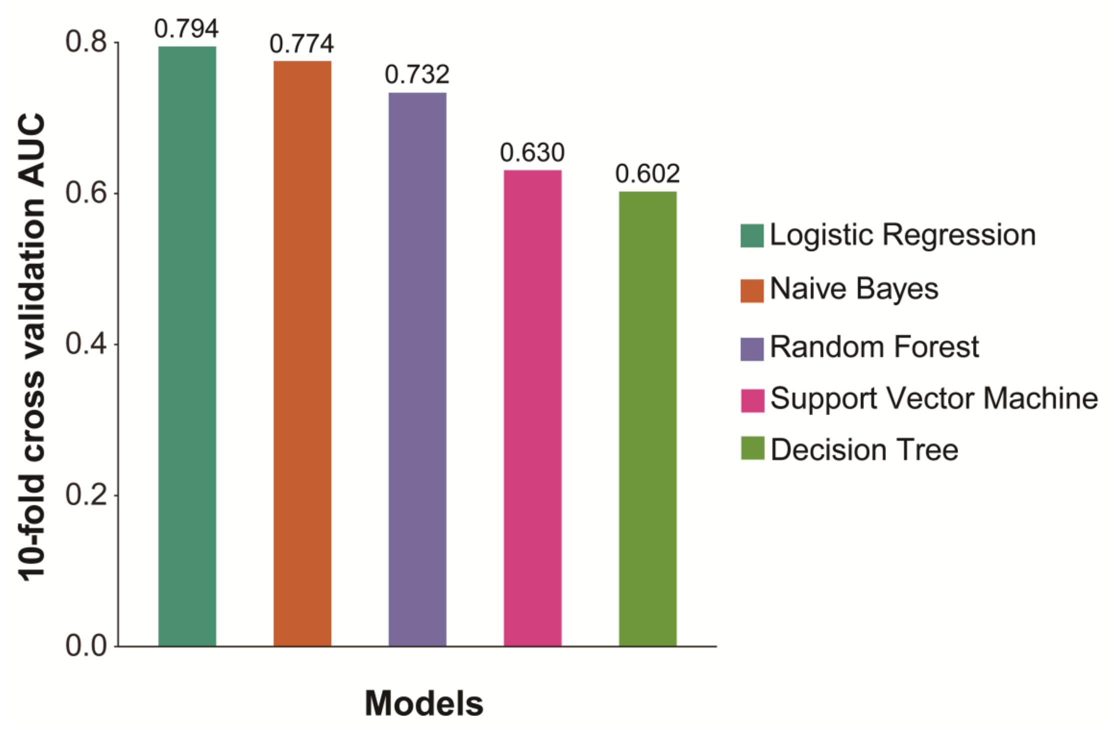

Figure S2. Performance of AUC for SEPPA 3.0 through 10-fold cross-validation.

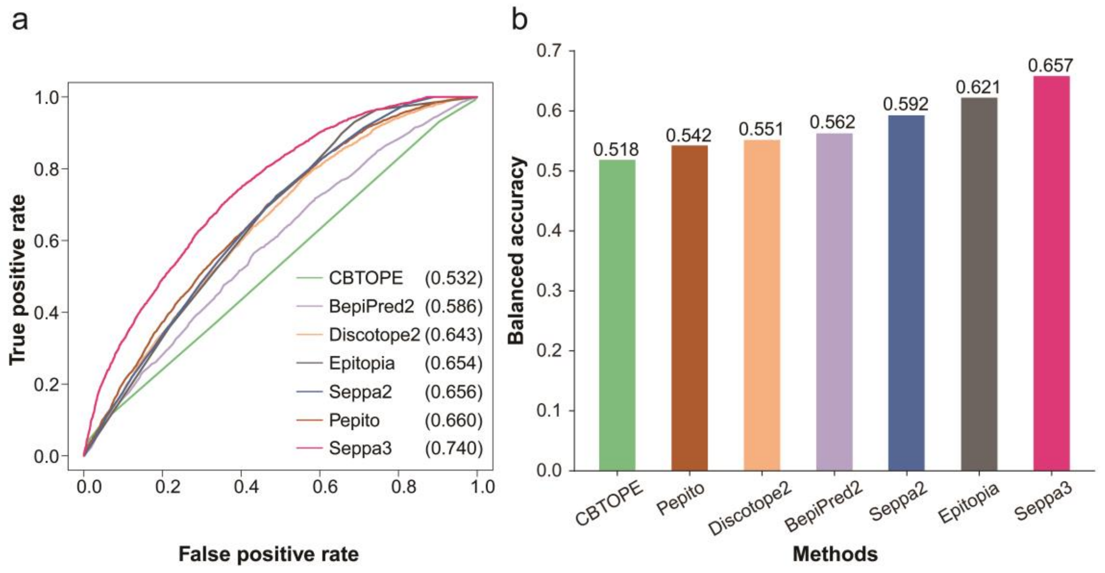

Figure S3. Performance comparison between SEPPA 3.0 and available peers. (a) ROC curves on independent testing of 130 general protein antigens. (b) Balanced accuracy on independent testing of 130 general protein antigens.

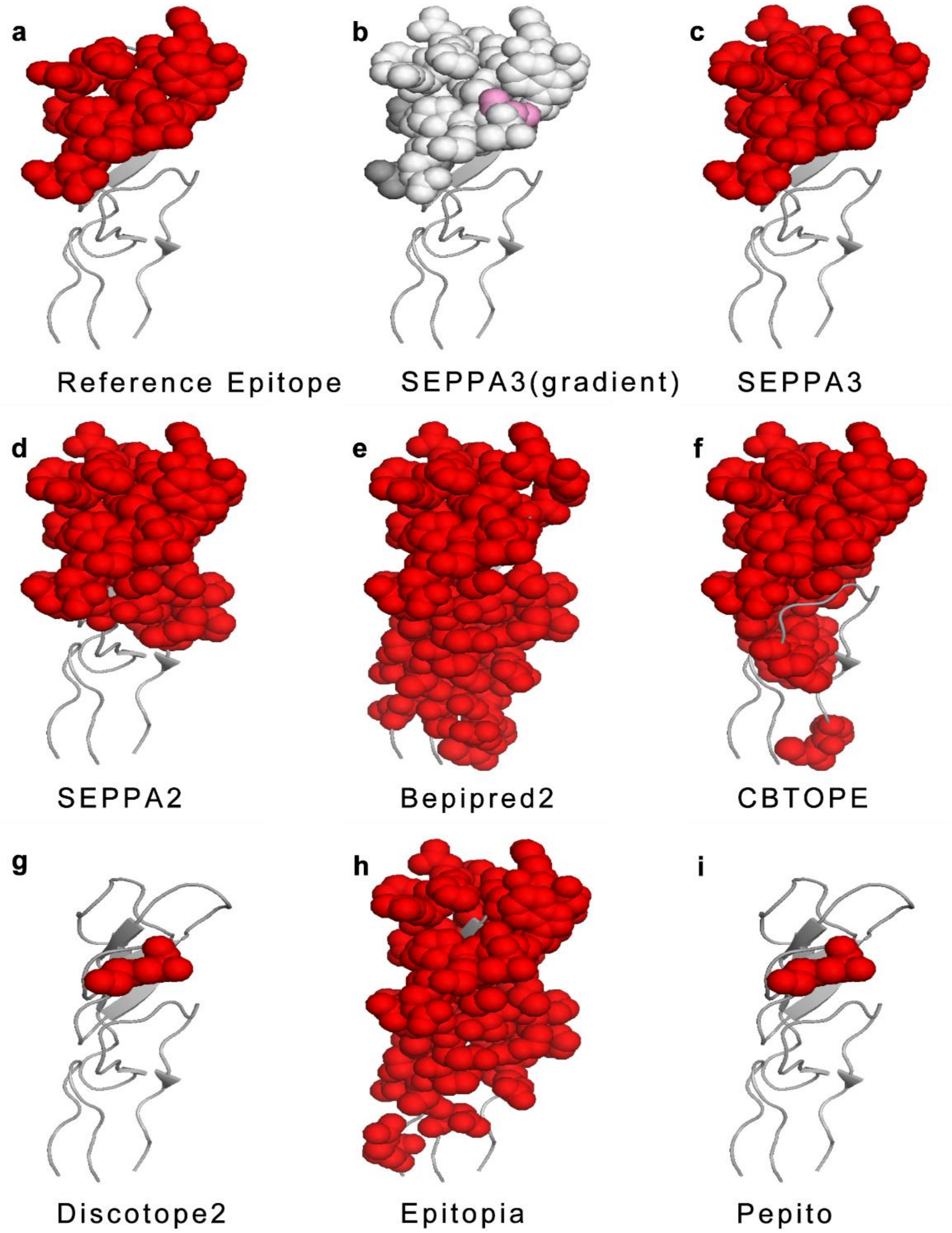

Figure S4. Case study of CD27. (a) Reference epitope of CD27 (PDB ID: 5TLK, Chain: X). (b) Predicted epitope residues for CD27 by SEPPA 3.0 with gradient illustration under default cutoff. Red, salmon, pink, white and grey color illustrate those potential candidates from high score to low score. (c) Predicted epitope residues for CD27 by SEPPA 3.0 with unified color under default cutoff. (d) Predicted epitope residues for CD27 by SEPPA 2.0. (e) Predicted epitope residues for CD27 by Bepipred-2.0. (f) Predicted epitope residues for CD27 by CBTOPE. (g) Predicted epitope residues for CD27 by Discotope-2.0. (h) Predicted epitope residues for CD27 by Epitopia. (i) Predicted epitope residues for CD27 by Pepito.

Supplementary Tables

Table S1. The training dataset and testing dataset of SEPPA 3.0*

Training dataset

No.	PDB id	Chain	No.	PDB id	Chain	No.	PDB id	Chain
**1**	1A14	N	**257**	3H3P	S	**513**	4L5F	E
**2**	1A2Y	C	**258**	3H42	A	**514**	4LAJ	J
**3**	1AFV	A	**259**	3H42	B	**515**	4LDE	A
**4**	1AHW	C	**260**	3HB3	B	**516**	4LDL	A
**5**	1AR1	B	**261**	3HFM	Y	**517**	4LDO	A
**6**	1BJ1	W	**262**	3HI1	G	**518**	4LEO	C
**7**	1BQL	Y	**263**	3HI6	A	**519**	4LF3	F
**8**	1BZQ	A	**264**	3HMX	A	**520**	4LHJ	A
**9**	1C08	C	**265**	3I50	E	**521**	4LIQ	E
**10**	1CZ8	W	**266**	3IDX	G	**522**	4LMQ	D
**11**	1DQJ	C	**267**	3IDY	G	**523**	4LQF	A
**12**	1DZB	X	**268**	3IU3	I	**524**	4LSP	G
**13**	1E6J	P	**269**	3IYW	A	**525**	4LSQ	G
**14**	1EGJ	A	**270**	3J1S	A	**526**	4LSR	G
**15**	1EO8	A	**271**	3J5M	A	**527**	4LSS	G
**16**	1EZV	E	**272**	3J70	D	**528**	4LST	G
**17**	1FBI	X	**273**	3J8V	E	**529**	4LSU	G
**18**	1FDL	Y	**274**	3J8W	E	**530**	4LSV	G
**19**	1FE8	A	**275**	3J8Z	E	**531**	4LU5	B
**20**	1FJ1	F	**276**	3JAB	C	**532**	4LVH	A
**21**	1FNS	A	**277**	3JBA	A	**533**	4LVN	A
**22**	1FSK	A	**278**	3JBQ	F	**534**	4LVO	A
**23**	1G6V	A	**279**	3JCB	A	**535**	4M1C	A
**24**	1G7H	C	**280**	3JCC	A	**536**	4M1G	A
**25**	1G7I	C	**281**	3JWD	A	**537**	4M3K	A
**26**	1G7J	C	**282**	3K2U	A	**538**	4M48	A
**27**	1G7L	C	**283**	3K7U	C	**539**	4M5Z	A
**28**	1G7M	C	**284**	3K80	C	**540**	4M62	S
**29**	1G9M	G	**285**	3K81	D	**541**	4M7L	T
**30**	1G9N	G	**286**	3KJ4	A	**542**	4M8Q	C
**31**	1GC1	G	**287**	3KJ6	A	**543**	4MA7	A
**32**	1H0D	C	**288**	3KR3	D	**544**	4MHH	C
**33**	1HEZ	E_1	**289**	3L5W	I	**545**	4MHJ	A
**34**	1HEZ	E_2	**290**	3L5X	A	**546**	4MWF	C
**35**	1HYS	B	**291**	3L95	Y	**547**	4MXV	A
**36**	1IC4	Y	**292**	3LD8	A	**548**	4MXW	A
**37**	1IC7	Y	**293**	3LH2	S	**549**	4N1H	A
**38**	1IGC	A	**294**	3LHP	S	**550**	4N90	S
**39**	1IQD	C	**295**	3LIZ	A	**551**	4N90	B

**40**	1J1O	Y	**296**	3LQA	G	**552**	4N9G	Y
**41**	1J1P	Y	**297**	3LZF	A	**553**	4NCO	A
**42**	1J5O	B	**298**	3MA9	A	**554**	4NHG	J
**43**	1JHL	A	**299**	3MAC	A	**555**	4NIK	A
**44**	1JPS	T	**300**	3MJ9	A	**556**	4NM8	A
**45**	1JRH	I	**301**	3MXW	A	**557**	4NM8	B
**46**	1JTO	L	**302**	3N85	A	**558**	4NNP	A
**47**	1JTP	L	**303**	3NCY	A	**559**	4NP4	A_1
**48**	1JTT	L	**304**	3NFP	I	**560**	4NP4	A_2
**49**	1K4C	C	**305**	3NH7	A	**561**	4NZR	M
**50**	1KEN	A	**306**	3NID	A	**562**	4NZT	M
**51**	1KIP	C	**307**	3O0R	B	**563**	4O02	A
**52**	1KIR	C	**308**	3O0R	C	**564**	4O58	A
**53**	1KXQ	A	**309**	3O2D	A	**565**	4O5I	A
**54**	1KXT	A	**310**	3OGC	C	**566**	4O9H	A
**55**	1KYO	E	**311**	3OPZ	A	**567**	4OD2	S
**56**	1LK3	A	**312**	3P0G	A	**568**	4ODX	X
**57**	1MEL	L	**313**	3P0Y	A	**569**	4OGX	A
**58**	1MHP	A	**314**	3P11	A	**570**	4OGY	A
**59**	1MLC	E	**315**	3P30	A	**571**	4OII	A
**60**	1MVF	E	**316**	3PGF	A	**572**	4OKV	F
**61**	1N5Y	B	**317**	3PJS	M	**573**	4ORZ	B
**62**	1N6Q	B	**318**	3PNW	C	**574**	4OT1	A
**63**	1N8Z	C	**319**	3Q1S	I	**575**	4P59	A
**64**	1NBY	C	**320**	3Q3G	E	**576**	4P9H	G
**65**	1NBZ	C	**321**	3QA3	E	**577**	4PGJ	B
**66**	1NCA	N	**322**	3QSK	A	**578**	4PLJ	A
**67**	1NCB	N	**323**	3QUM	P_2	**579**	4PLK	B
**68**	1NCC	N	**324**	3QUM	P_1	**580**	4PP1	B
**69**	1NDG	C	**325**	3QWO	P	**581**	4PP2	F
**70**	1NDM	C	**326**	3R08	E	**582**	4PS4	A
**71**	1NL0	G	**327**	3R1G	B	**583**	4PY8	A
**72**	1NMA	N	**328**	3RAJ	A	**584**	4PY8	B
**73**	1NMB	N	**329**	3RHW	A	**585**	4Q5Z	A
**74**	1NSN	S	**330**	3RKD	A	**586**	4Q6I	J
**75**	1OAK	A	**331**	3RU8	X	**587**	4QCI	C
**76**	1OAZ	A	**332**	3RVV	A	**588**	4QEX	A
**77**	1OB1	C	**333**	3RVW	A	**589**	4QHU	C
**78**	1ORQ	C	**334**	3S35	X	**590**	4QNP	B
**79**	1ORS	C	**335**	3S36	X	**591**	4QTI	U
**80**	1OSP	O	**336**	3S37	X	**592**	4QWW	A
**81**	1OTS	A	**337**	3SDY	A	**593**	4R0L	D
**82**	1OTT	A	**338**	3SDY	B	**594**	4R2G	O
**83**	1P2C	C	**339**	3SE8	G	**595**	4R4F	A

**84**	1QFU	A	**340**	3SE9	G	**596**	4R4H	A
**85**	1QFW	A	**341**	3SKJ	E	**597**	4R4N	A
**86**	1QLE	B	**342**	3SN6	A	**598**	4R8W	A
**87**	1R3J	C	**343**	3SOB	B	**599**	4R8W	B
**88**	1RI8	B	**344**	3SQO	A	**600**	4R9Y	B
**89**	1RJC	B	**345**	3STB	C	**601**	4RAU	C
**90**	1RJL	C	**346**	3STB	D	**602**	4RDQ	A
**91**	1S78	A	**347**	3T2N	A	**603**	4RFN	G
**92**	1SY6	A	**348**	3THM	F	**604**	4RFO	G
**93**	1TPX	A	**349**	3TJE	F	**605**	4RGM	S
**94**	1TQC	A	**350**	3TT1	B	**606**	4RGN	S_1
**95**	1TZH	V	**351**	3TT3	A	**607**	4RGN	S_2
**96**	1TZI	V	**352**	3TYG	A	**608**	4RGO	S
**97**	1UA6	Y	**353**	3U2S	G	**609**	4RQS	G
**98**	1UAC	Y	**354**	3U30	A	**610**	4RRP	R
**99**	1UJ3	C	**355**	3U4E	G	**611**	4RWY	A
**100**	1V7M	V	**356**	3U7Y	G	**612**	4RX4	G
**101**	1V7N	V	**357**	3U9P	D	**613**	4S1Q	G
**102**	1VFB	C	**358**	3U9U	F	**614**	4S1R	G
**103**	1W72	A	**359**	3UAJ	A	**615**	4S1S	G
**104**	1WEJ	F	**360**	3UBX	A	**616**	4TSA	A
**105**	1XCQ	L	**361**	3UC0	A	**617**	4TSB	A
**106**	1XFP	L	**362**	3ULU	A_2	**618**	4TSC	A
**107**	1XGP	C	**363**	3ULU	A_1	**619**	4U0R	A
**108**	1XGQ	C	**364**	3ULU	A_3	**620**	4U1G	D
**109**	1XGT	C	**365**	3ULV	A_1	**621**	4U3X	B
**110**	1XIW	B	**366**	3ULV	A_2	**622**	4U6H	E
**111**	1XIW	E	**367**	3ULV	A_3	**623**	4UAO	A
**112**	1YJD	C	**368**	3UX9	A	**624**	4UBD	A
**113**	1YMH	E	**369**	3V0A	A	**625**	4UBD	B
**114**	1YNT	F	**370**	3V4P	B	**626**	4UT6	A
**115**	1YNT	E	**371**	3V4V	B	**627**	4UT9	A
**116**	1YQV	Y	**372**	3V6O	A	**628**	4UTA	B
**117**	1YY9	A	**373**	3V6Z	F	**629**	4UTB	A
**118**	1Z3G	A	**374**	3V7A	B	**630**	4V1D	C_1
**119**	1ZA3	R	**375**	3VE0	I	**631**	4V1D	C_2
**120**	1ZMY	L	**376**	3VE0	J	**632**	4WEB	E
**121**	1ZTX	E	**377**	3VG9	A	**633**	4WFE	A
**122**	1ZV5	L	**378**	3VGA	A	**634**	4WGV	A
**123**	1ZVH	L	**379**	3VI3	D	**635**	4WV1	C
**124**	1ZVY	B	**380**	3VI4	D	**636**	4XAK	A
**125**	2A0L	A	**381**	3VRL	C	**637**	4XHJ	A
**126**	2ADF	A	**382**	3W11	E	**638**	4XHJ	B
**127**	2AEP	A	**383**	3W13	E	**639**	4XI5	A

**128**	2AEQ	A	**384**	3W14	E	**640**	4XI5	B
**129**	2ATK	C	**385**	3W2D	A	**641**	4XMM	D
**130**	2B2X	A	**386**	3W9E	C	**642**	4XMN	E
**131**	2B4C	G	**387**	3WD5	A	**643**	4XMP	G
**132**	2BDN	A	**388**	3WIH	A	**644**	4XNM	C
**133**	2BSE	A	**389**	3WKM	A	**645**	4XNQ	C
**134**	2DD8	S	**390**	3WLW	B	**646**	4XNU	A
**135**	2DQC	Y	**391**	3WSQ	A	**647**	4XNX	A
**136**	2DQD	Y	**392**	3X3F	A	**648**	4XNY	G
**137**	2DQE	Y	**393**	3ZKM	A	**649**	4XNZ	G
**138**	2DQF	C	**394**	3ZKN	A	**650**	4XP4	A
**139**	2DQG	Y	**395**	3ZTJ	A	**651**	4XP6	A
**140**	2EIZ	C	**396**	3ZTJ	B	**652**	4XPA	A
**141**	2EXW	A	**397**	3ZTN	A	**653**	4XPB	A
**142**	2FD6	U	**398**	3ZTN	B	**654**	4XPG	A
**143**	2FEC	A	**399**	4AEI	A	**655**	4XPH	A
**144**	2FJG	V	**400**	4AG4	A	**656**	4XPT	A
**145**	2FJH	V	**401**	4AL8	C	**657**	4XRC	C
**146**	2GHW	A	**402**	4ALA	C	**658**	4XTR	B
**147**	2H2P	A	**403**	4AM0	R	**659**	4XVS	G
**148**	2H2S	A	**404**	4BZ1	A	**660**	4XVT	G
**149**	2H3N	A	**405**	4BZ2	A	**661**	4YBL	A
**150**	2H9G	S	**406**	4CAD	C	**662**	4YBQ	B
**151**	2HFG	R	**407**	4CKD	C	**663**	4YC2	A
**152**	2HMI	B	**408**	4CMH	A	**664**	4YDI	G
**153**	2I9L	I	**409**	4CNI	C	**665**	4YDJ	G
**154**	2J4W	D	**410**	4D3C	A	**666**	4YDK	G
**155**	2J5L	A	**411**	4D9Q	A	**667**	4YDL	G
**156**	2J88	A	**412**	4D9R	A	**668**	4YE4	G
**157**	2JEL	P	**413**	4DAG	A	**669**	4YFL	G
**158**	2JIX	B	**414**	4DGI	A	**670**	4YJZ	E
**159**	2NLJ	C	**415**	4DK3	C	**671**	4YK4	A
**160**	2NR6	A	**416**	4DK6	D	**672**	4YPG	D
**161**	2NY1	A	**417**	4DKA	C	**673**	4YUE	C
**162**	2NY3	A	**418**	4DKE	A	**674**	4YWG	G
**163**	2NY5	G	**419**	4DKF	A	**675**	4YX2	A
**164**	2NY7	G	**420**	4DN4	M	**676**	4YXH	A
**165**	2NYY	A	**421**	4DQO	C	**677**	4YXK	A
**166**	2OZ4	A	**422**	4DTG	K	**678**	4YXL	A
**167**	2P42	A	**423**	4DVR	G	**679**	4YZF	A
**168**	2P43	A	**424**	4DW2	U	**680**	4Z5R	N
**169**	2P44	A	**425**	4EDW	V	**681**	4Z9K	A
**170**	2P45	A	**426**	4EDX	V	**682**	4ZFO	K
**171**	2P46	A	**427**	4EIG	A	**683**	4ZPT	S

**172**	2P4A	A	**428**	4ERS	A	**684**	4ZPV	R
**173**	2Q8A	A	**429**	4F15	D	**685**	4ZS6	A
**174**	2Q8B	A	**430**	4F2M	E	**686**	4ZS7	A
**175**	2QAD	A	**431**	4F37	A	**687**	4ZXB	E_1
**176**	2QQK	A	**432**	4F37	H	**688**	4ZXB	E_2
**177**	2QQL	A	**433**	4F3F	C	**689**	4ZYP	C_1
**178**	2QQN	A	**434**	4F9L	A	**690**	4ZYP	C_2
**179**	2QR0	C	**435**	4F9P	A	**691**	5A3I	E
**180**	2R0K	A	**436**	4FFV	A	**692**	5A7X	A
**181**	2R0L	A	**437**	4FFW	A	**693**	5A8H	A
**182**	2R29	A	**438**	4FFY	A	**694**	5AAM	C
**183**	2R4R	A	**439**	4FFZ	A	**695**	5AAW	C
**184**	2R4S	A	**440**	4FP8	A	**696**	5ACO	A
**185**	2R56	A	**441**	4FQI	A	**697**	5ANY	B
**186**	2R69	A	**442**	4FQI	B	**698**	5BO1	B
**187**	2UZI	R	**443**	4FQJ	A	**699**	5BOZ	A
**188**	2VC2	A	**444**	4FQK	A	**700**	5BV7	A_1
**189**	2VIR	C	**445**	4FQR	A	**701**	5BV7	A_2
**190**	2VIS	C	**446**	4FQV	A	**702**	5BVP	I
**191**	2VIT	C	**447**	4FQV	B	**703**	5C0N	A
**192**	2VWE	A	**448**	4FQY	A	**704**	5C0R	A
**193**	2VXQ	A	**449**	4FQY	B	**705**	5C0S	A
**194**	2VXT	I	**450**	4G3Y	C	**706**	5C6T	A
**195**	2VYR	A	**451**	4G6J	A	**707**	5C7K	C_2
**196**	2W9E	A	**452**	4G6M	A	**708**	5C7K	C_1
**197**	2WUB	A	**453**	4G7V	S	**709**	5C7K	D
**198**	2WUC	A	**454**	4G7Y	S	**710**	5C7X	A
**199**	2X7L	N	**455**	4G80	T	**711**	5C8J	I
**200**	2X89	D	**456**	4GMS	A	**712**	5CD5	A
**201**	2XQB	A	**457**	4GXU	A	**713**	5CEZ	B
**202**	2XQY	A	**458**	4H88	A	**714**	5CEZ	G
**203**	2XRA	A	**459**	4H8W	G	**715**	5CJO	A
**204**	2XWT	C	**460**	4HC1	A	**716**	5CJX	K
**205**	2YBR	C	**461**	4HCR	A	**717**	5CUS	A
**206**	2YPV	A	**462**	4HF5	A	**718**	5CWS	D
**207**	2YSS	C	**463**	4HFU	A	**719**	5CZV	A
**208**	2ZCH	P	**464**	4HG4	A	**720**	5CZX	A
**209**	2ZCK	P	**465**	4HGK	B	**721**	5D1Q	E_2
**210**	2ZCL	P	**466**	4HJ0	A	**722**	5D1Q	E_1
**211**	2ZJS	Y	**467**	4HJG	H	**723**	5D1X	E_1
**212**	2ZNX	Y	**468**	4HJG	E	**724**	5D1X	E_2
**213**	2ZUQ	A	**469**	4HKX	E	**725**	5D1Z	I_1
**214**	3A67	Y	**470**	4HKZ	E	**726**	5D1Z	I_2
**215**	3A6B	Y	**471**	4HLZ	B	**727**	5D70	A

**216**	3AB0	A	**472**	4HLZ	A	**728**	5D72	A
**217**	3B2U	A	**473**	4HWB	A	**729**	5D8J	A
**218**	3B2V	A	**474**	4I0C	B	**730**	5D93	A
**219**	3B9K	B	**475**	4I18	C	**731**	5D96	A
**220**	3BDY	V	**476**	4I3R	G	**732**	5D9Q	A_2
**221**	3BE1	A	**477**	4I3S	G	**733**	5D9Q	A_1
**222**	3BGF	S	**478**	4I77	Z	**734**	5DFV	A
**223**	3BN9	B	**479**	4I9W	A	**735**	5DFW	A
**224**	3BSZ	F	**480**	4IDJ	A	**736**	5DHV	M
**225**	3BT2	U	**481**	4IJ3	A	**737**	5DMI	A
**226**	3C09	D	**482**	4IOF	A	**738**	5DMJ	A
**227**	3CSY	J	**483**	4IRZ	A	**739**	5DO2	A
**228**	3CVH	A	**484**	4J6R	G	**740**	5DUM	A
**229**	3CXH	E	**485**	4JB9	G	**741**	5DUP	A
**230**	3D85	C	**486**	4JDT	G	**742**	5DUR	C
**231**	3D9A	C	**487**	4JHW	F	**743**	5DWU	A
**232**	3DVG	X	**488**	4JKP	G	**744**	5DWU	B
**233**	3DVG	Y	**489**	4JM2	E_2	**745**	5E1A	C
**234**	3DVN	U	**490**	4JM2	E_1	**746**	5E1H	A
**235**	3EFF	M	**491**	4JPK	A	**747**	5E8D	A
**236**	3EHB	B	**492**	4JPV	G	**748**	5E94	G
**237**	3EO1	C	**493**	4JPW	G	**749**	5EII	I
**238**	3EOA	I	**494**	4JZJ	C	**750**	5EN2	C
**239**	3EOB	I	**495**	4K24	U	**751**	5ESV	G
**240**	3ETB	J	**496**	4K2U	A	**752**	5ESZ	G
**241**	3F7V	C	**497**	4K3J	B	**753**	5EU7	A
**242**	3FB5	C	**498**	4K8R	B_1	**754**	5EZO	H
**243**	3FFD	P	**499**	4K8R	B_2	**755**	5F3B	C
**244**	3FKU	F	**500**	4K94	C	**756**	5F3J	A
**245**	3FMG	A	**501**	4K9E	C	**757**	5F6J	G
**246**	3G04	C	**502**	4KHT	A	**758**	5F72	K
**247**	3G6D	A	**503**	4KHX	A	**759**	5F96	G
**248**	3G6J	B	**504**	4KI5	M_1	**760**	5F9O	G
**249**	3GBM	A	**505**	4KI5	M_2	**761**	5F9W	G
**250**	3GBM	B	**506**	4KKC	B	**762**	5FB8	C
**251**	3GBN	A	**507**	4KRO	A	**763**	5FCU	G
**252**	3GBN	B	**508**	4KRP	A	**764**	5FEC	I
**253**	3GI8	C	**509**	4KUC	A	**765**	5FHC	K
**254**	3GI9	C	**510**	4KV5	A	**766**	5FHC	J
**255**	3GRW	A	**511**	4KVN	A	**767**	5FHX	A
**256**	3H3B	A	**512**	4KXZ	E			

Testing dataset

Glycoprotein antigen

No.	PDB id	Chain	No.	PDB id	Chain	No.	PDB id	Chain
**1**	3JCX	A	**37**	5J3D	K	**73**	5MVZ	U
**2**	5B3J	D	**38**	5J3H	E	**74**	5SV3	B
**3**	5B3J	A	**39**	5J56	A	**75**	5SX4	N
**4**	5B8C	C	**40**	5J57	A	**76**	5SX5	N
**5**	5FUU	F	**41**	5JQ6	A	**77**	5T33	G
**6**	5FUU	C	**42**	5JS9	D	**78**	5T3S	G
**7**	5FV1	V	**43**	5JS9	C	**79**	5T3X	G_1
**8**	5FV2	X	**44**	5JSA	D	**80**	5T3X	G_2
**9**	5FYL	B	**45**	5JW3	B	**81**	5T3Z	G
**10**	5FYL	G	**46**	5JW3	A	**82**	5TE4	G
**11**	5GGR	Z	**47**	5JW4	A	**83**	5TE6	G
**12**	5GGS	Z	**48**	5JXE	B	**84**	5TE7	G
**13**	5GGV	Y	**49**	5JYL	A	**85**	5TH9	A
**14**	5GJS	B	**50**	5JYM	A	**86**	5THR	C
**15**	5GJS	A	**51**	5JZ7	B	**87**	5THR	D
**16**	5GJT	B	**52**	5K59	A	**88**	5TLJ	X_1
**17**	5GJT	A	**53**	5K9K	I	**89**	5TLJ	X_2
**18**	5GRJ	A	**54**	5K9O	I	**90**	5TLK	X_1
**19**	5GS0	A	**55**	5K9Q	F	**91**	5TLK	X_2
**20**	5GZN	A	**56**	5KAN	D	**92**	5TOJ	A
**21**	5H37	A	**57**	5KAN	C	**93**	5TOK	B
**22**	5HDQ	A	**58**	5KAQ	B	**94**	5TPN	A
**23**	5HHX	A	**59**	5KEL	B	**95**	5TPW	A
**24**	5HI3	B	**60**	5KEL	A	**96**	5TQ0	A
**25**	5HI4	A	**61**	5KEM	A_1	**97**	5TQ2	A
**26**	5HI5	A	**62**	5KEM	A_2	**98**	5TQQ	A
**27**	5HJ3	D	**63**	5KEN	B	**99**	5TR1	A
**28**	5I6X	A	**64**	5KEN	E	**100**	5TZ2	C
**29**	5I6Z	A	**65**	5KJR	G	**101**	5TZT	C
**30**	5I74	A	**66**	5KOV	A	**102**	5TZU	C
**31**	5I8H	B	**67**	5KTE	A	**103**	5U1F	A
**32**	5I8H	C	**68**	5KW9	A	**104**	5UGY	A
**33**	5I9Q	G	**69**	5KZC	A	**105**	5UQY	E
**34**	5IES	C	**70**	5L0Q	A	**106**	5WT9	G
**35**	5IF0	G	**71**	5L6Y	C			
**36**	5IGX	G	**72**	5M94	A			

Non-glycoprotein antigen

No.	PDB id	Chain	No.	PDB id	Chain	No.	PDB id	Chain
**107**	5FUO	A	**115**	5KVD	E	**123**	5SY8	O
**108**	5GS1	D	**116**	5KVE	E	**124**	5T29	O
**109**	5GZO	A	**117**	5KVF	E	**125**	5T5B	E

**110**	5H35	C	**118**	5KVG	E	**126**	5T5F	A
**111**	5HGG	B	**119**	5LQB	A	**127**	5T85	G
**112**	5HHV	A	**120**	5MES	A	**128**	5TFW	O
**113**	5IHL	A	**121**	5MEV	A	**129**	5TIH	A
**114**	5JHL	A	**122**	5MI0	A	**130**	5WTH	B

*For one protein antigen contains multiple epitope regions, they will be marked as chain name+number, such as A_1, A_2.

Table S2. The performance of SEPPA 3.0 compared with available peers

**General Protein antigen***	**Threshold**	**AUC**	**BA[a]**	**FPR[b]**
**CBTOPE**	4.000	0.532	0.518	0.332
**Bepipred2.0**	0.500	0.586	0.562	0.458
**Discotope2.0**	-3.700	0.643	0.551	0.129
**Epitopia**	0.065	0.654	0.621	0.655
**Pepito**	1.300	0.660	0.542	0.082
**SEPPA2.0**	0.100	0.656	0.592	0.297
**Raw_SEPPA3.0**	0.069	0.728	0.662	0.350
**SEPPA 3.0**	0.089	0.740	0.657	0.258
**Glycoprotein antigen#**	**Threshold**	**AUC**	**BA**	**FPR**
**CBTOPE**	4.000	0.541	0.524	0.339
**Bepipred2.0**	0.500	0.591	0.566	0.467
**Discotope2.0**	-3.700	0.660	0.564	0.135
**Epitopia**	0.065	0.664	0.630	0.648
**Pepito**	1.300	0.676	0.551	0.085
**SEPPA 2.0**	0.100	0.651	0.589	0.286
**Raw_SEPPA 3.0**	0.069	0.736	0.667	0.340
**SEPPA 3.0**	0.089	0.749	0.665	0.252

*Prediction performance of general protein antigen

#Prediction performance of glycoprotein antigen

[a]BA represents Balanced Accuracy

[b]FPR represents False Positive Rate

Table S3. Performance (AUC) of individual structures from SEPPA 3.0 and available peers.

**Glycoprotein antigen**								
**No.**	PDB id	SEPPA3.0	SEPPA2.0	Pepito	Epitopia	Discotope2.0	CBTOPE	Bepipred2.0
**1**	3JCX_A	0.681	0.551	0.764	0.763	0.725	0.532	0.546
**2**	5B3J_A	0.812	0.491	0.783	0.774	0.796	0.476	0.701
**3**	5B3J_D	0.788	0.523	0.645	0.777	0.654	0.479	0.623
**4**	5B8C_C	0.664	0.651	0.659	0.549	0.723	0.543	0.628
**5**	5FUU_C	0.704	0.582	0.459	0.687	0.584	0.509	0.660
**6**	5FUU_F	0.654	0.565	0.592	0.697	0.716	0.500	0.836
**7**	5FV1_V	0.554	0.498	0.596	0.683	0.704	0.722	0.636

**8**	5FV2_X	0.643	0.509	0.605	0.731	0.709	0.635	0.595
**9**	5FYL_G	0.870	0.702	0.768	0.845	0.772	0.493	0.618
**10**	5FYL_B	0.606	0.471	0.656	0.759	0.610	0.488	0.736
**11**	5GGR_Z	0.984	0.968	0.803	0.453	0.895	0.593	0.815
**12**	5GGS_Z	0.696	0.718	0.639	0.542	0.702	0.524	0.573
**13**	5GGV_Y	0.538	0.629	0.641	0.535	0.641	0.484	0.627
**14**	5GJS_A	0.646	0.573	0.788	0.647	0.840	0.528	0.541
**15**	5GJS_B	0.901	0.647	0.572	0.534	0.522	0.442	0.549
**16**	5GJT_A	0.685	0.597	0.700	0.630	0.738	0.563	0.622
**17**	5GJT_B	0.817	0.624	0.563	0.571	0.508	0.439	0.525
**18**	5GRJ_A	0.658	0.593	0.465	0.521	0.546	0.546	0.570
**19**	5GS0_A	0.816	0.385	0.673	0.713	0.576	0.647	0.675
**20**	5GZN_A	0.530	0.618	0.766	0.624	0.740	0.560	0.503
**21**	5H37_A	0.920	0.702	0.605	0.701	0.728	0.703	0.658
**22**	5HDQ_A	0.602	0.864	0.484	0.858	0.554	0.430	0.521
**23**	5HHX_A	0.760	0.491	0.712	0.738	0.793	0.630	0.482
**24**	5HI3_B	0.923	0.641	0.703	0.689	0.788	0.675	0.467
**25**	5HI4_A	0.657	0.543	0.664	0.644	0.706	0.679	0.480
**26**	5HI5_A	0.910	0.869	0.883	0.448	0.833	0.259	0.905
**27**	5HJ3_D	0.527	0.742	0.599	0.713	0.834	0.954	0.765
**28**	5I6X_A	0.603	0.615	0.925	0.824	0.932	0.684	0.846
**29**	5I6Z_A	0.754	0.540	0.913	0.825	0.912	0.752	0.841
**30**	5I74_A	0.724	0.669	0.921	0.829	0.930	0.728	0.843
**31**	5I8H_C	0.895	0.586	0.767	0.738	0.769	0.508	0.636
**32**	5I8H_B	0.612	0.798	0.465	0.782	0.618	0.805	0.715
**33**	5I9Q_G	0.834	0.682	0.776	0.585	0.798	0.512	0.723
**34**	5IES_C	0.857	0.861	0.878	0.667	0.885	0.614	0.766
**35**	5IF0_G	0.936	0.841	0.885	0.645	0.895	0.645	0.773
**36**	5IGX_G	0.866	0.780	0.821	0.690	0.863	0.513	0.690
**37**	5J3D_K	0.693	0.784	0.760	0.798	0.792	0.830	0.631
**38**	5J3H_E	0.939	0.530	0.609	0.741	0.634	0.810	0.639
**39**	5J56_A	0.865	0.860	0.542	0.732	0.505	0.366	0.566
**40**	5J57_A	0.677	0.781	0.747	0.829	0.734	0.601	0.689
**41**	5JQ6_A	0.504	0.690	0.780	0.695	0.755	0.546	0.552
**42**	5JS9_C	0.863	0.504	0.721	0.831	0.670	0.374	0.641
**43**	5JS9_D	0.859	0.466	0.857	0.843	0.773	0.444	0.520
**44**	5JSA_D	0.961	0.586	0.871	0.838	0.801	0.484	0.560
**45**	5JW3_A	0.904	0.838	0.737	0.640	0.757	0.433	0.489
**46**	5JW3_B	0.702	0.583	0.525	0.579	0.615	0.450	0.486
**47**	5JW4_A	0.803	0.562	0.729	0.793	0.798	0.633	0.534
**48**	5JXE_B	0.546	0.722	0.686	0.577	0.772	0.498	0.592
**49**	5JYL_A	0.839	0.853	0.750	0.610	0.725	0.447	0.745
**50**	5JYM_A	0.542	0.724	0.613	0.592	0.599	0.581	0.535
**51**	5JZ7_B	0.653	0.678	0.527	0.579	0.667	0.454	0.551

**52**	5K59_A	0.733	0.735	0.802	0.764	0.731	0.463	0.659
**53**	5K9K_I	0.603	0.659	0.565	0.674	0.616	0.390	0.582
**54**	5K9O_I	0.627	0.506	0.565	0.477	0.588	0.493	0.592
**55**	5K9Q_F	0.883	0.569	0.646	0.716	0.552	0.296	0.642
**56**	5KAN_C	0.539	0.569	0.618	0.789	0.591	0.634	0.716
**57**	5KAN_D	0.922	0.539	0.637	0.615	0.532	0.283	0.662
**58**	5KAQ_B	0.875	0.466	0.627	0.561	0.654	0.313	0.690
**59**	5KEL_B	0.695	0.464	0.567	0.758	0.757	0.951	0.779
**60**	5KEL_A	0.570	0.546	0.760	0.733	0.823	0.464	0.611
**61**	5KEM_A_1	0.672	0.801	0.538	0.513	0.691	0.508	0.608
**62**	5KEM_A_2	0.565	0.604	0.688	0.719	0.784	0.429	0.568
**63**	5KEN_E	0.661	0.567	0.723	0.743	0.774	0.502	0.521
**64**	5KEN_B	0.650	0.441	0.645	0.748	0.807	0.910	0.726
**65**	5KJR_G	0.570	0.559	0.646	0.571	0.641	0.390	0.659
**66**	5KOV_A	0.628	0.783	0.685	0.770	0.679	0.548	0.603
**67**	5KTE_A	0.735	0.725	0.733	0.832	0.758	0.711	0.805
**68**	5KW9_A	0.684	0.624	0.847	0.683	0.897	0.588	0.618
**69**	5KZC_A	0.709	0.783	0.881	0.622	0.885	0.579	0.749
**70**	5L0Q_A	0.682	0.445	0.663	0.574	0.602	0.410	0.557
**71**	5L6Y_C	0.713	0.694	0.775	0.464	0.785	0.547	0.540
**72**	5M94_A	0.897	0.751	0.615	0.788	0.593	0.549	0.800
**73**	5MVZ_U	0.727	0.609	0.594	0.562	0.542	0.769	0.556
**74**	5SV3_B	0.841	0.815	0.761	0.597	0.715	0.508	0.652
**75**	5SX4_N	0.845	0.728	0.586	0.595	0.604	0.915	0.655
**76**	5SX5_N	0.868	0.618	0.628	0.672	0.630	0.914	0.657
**77**	5T33_G	0.909	0.874	0.900	0.712	0.928	0.403	0.810
**78**	5T3S_G	0.776	0.524	0.606	0.668	0.576	0.398	0.619
**79**	5T3X_G_1	0.914	0.685	0.732	0.815	0.716	0.428	0.573
**80**	5T3X_G_2	0.601	0.553	0.771	0.563	0.720	0.530	0.575
**81**	5T3Z_G	0.615	0.542	0.771	0.529	0.730	0.494	0.591
**82**	5TE4_G	0.886	0.662	0.777	0.562	0.767	0.377	0.623
**83**	5TE6_G	0.951	0.863	0.787	0.543	0.801	0.568	0.622
**84**	5TE7_G	0.935	0.763	0.772	0.566	0.764	0.390	0.607
**85**	5TH9_A	0.716	0.889	0.777	0.799	0.761	0.349	0.590
**86**	5THR_C	0.634	0.671	0.783	0.683	0.722	0.474	0.515
**87**	5THR_D	0.908	0.849	0.502	0.622	0.446	0.708	0.525
**88**	5TLJ_X_1	0.829	0.823	0.924	0.778	0.972	0.755	0.698
**89**	5TLJ_X_2	0.673	0.699	0.661	0.627	0.751	0.467	0.557
**90**	5TLK_X_1	0.960	0.835	0.931	0.745	0.970	0.792	0.727
**91**	5TLK_X_2	0.827	0.640	0.728	0.521	0.813	0.474	0.582
**92**	5TOJ_A	0.647	0.590	0.459	0.605	0.536	0.563	0.647
**93**	5TOK_B	0.561	0.600	0.575	0.647	0.500	0.579	0.641
**94**	5TPN_A	0.616	0.475	0.525	0.611	0.507	0.464	0.719
**95**	5TPW_A	0.835	0.459	0.586	0.653	0.569	0.495	0.642

**96**	5TQ0_A	0.854	0.546	0.543	0.615	0.548	0.489	0.627
**97**	5TQ2_A	0.812	0.510	0.539	0.541	0.532	0.501	0.694
**98**	5TQQ_A	0.602	0.774	0.815	0.735	0.833	0.519	0.765
**99**	5TR1_A	0.567	0.710	0.833	0.750	0.853	0.520	0.790
**100**	5TZ2_C	0.785	0.571	0.613	0.728	0.540	0.447	0.507
**101**	5TZT_C	0.689	0.728	0.514	0.727	0.473	0.458	0.460
**102**	5TZU_C	0.479	0.676	0.570	0.561	0.514	0.409	0.522
**103**	5U1F_A	0.911	0.882	0.884	0.953	0.824	0.375	0.575
**104**	5UGY_A	0.899	0.610	0.771	0.642	0.849	0.492	0.559
**105**	5UQY_E	0.571	0.525	0.600	0.559	0.434	0.572	0.662
**106**	5WT9_G	0.948	0.980	0.884	0.473	0.948	0.610	0.839
	Average [a]	0.743	0.655	0.694	0.671	0.710	0.546	0.637

Non-glycoprotein antigen

No.	PDB id	SEPPA3.0	SEPPA2.0	Pepito	Epitopia	Discotope2.0	CBTOPE	Bepipred2.0
**107**	5FUO_A	0.521	0.510	0.855	0.666	0.774	0.627	0.611
**108**	5GS1_D	0.702	0.422	0.543	0.651	0.552	0.560	0.540
**109**	5GZO_A	0.650	0.538	0.503	0.615	0.490	0.642	0.545
**110**	5H35_C	0.651	0.789	0.654	0.693	0.624	0.552	0.644
**111**	5HGG_B	0.752	0.735	0.518	0.541	0.551	0.551	0.599
**112**	5HHV_A	0.828	0.559	0.645	0.673	0.689	0.721	0.568
**113**	5IHL_A	0.764	0.737	0.666	0.712	0.773	0.630	0.554
**114**	5JHL_A	0.997	0.896	0.803	0.791	0.900	0.755	0.796
**115**	5KVD_E	0.637	0.641	0.597	0.698	0.581	0.520	0.499
**116**	5KVE_E	0.552	0.763	0.548	0.550	0.532	0.452	0.521
**117**	5KVF_E	0.524	0.788	0.533	0.580	0.488	0.555	0.522
**118**	5KVG_E	0.572	0.517	0.556	0.703	0.552	0.471	0.664
**119**	5LQB_A	0.524	0.584	0.870	0.704	0.923	0.499	0.805
**120**	5MES_A	0.629	0.688	0.638	0.732	0.667	0.616	0.709
**121**	5MEV_A	0.647	0.638	0.675	0.680	0.690	0.595	0.707
**122**	5MI0_A	0.611	0.525	0.670	0.533	0.806	0.691	0.462
**123**	5SY8_O	0.827	0.627	0.562	0.564	0.511	0.470	0.554
**124**	5T29_O	0.829	0.684	0.530	0.506	0.525	0.472	0.520
**125**	5T5B_E	0.657	0.726	0.515	0.501	0.556	0.439	0.490
**126**	5T5F_A	0.624	0.681	0.924	0.818	0.981	0.642	0.538
**127**	5T85_G	0.731	0.715	0.570	0.569	0.525	0.487	0.508
**128**	5TFW_O	0.694	0.724	0.530	0.624	0.538	0.442	0.520
**129**	5TIH_A	0.847	0.738	0.725	0.744	0.669	0.517	0.557
**130**	5WTH_B	0.789	0.830	0.597	0.623	0.659	0.747	0.581
	Average [b]	0.733	0.657	0.683	0.666	0.699	0.550	0.627

[a] Averaged AUC value of 106 glycoprotein antigens.

[b] Averaged AUC value of 130 general protein antigens.

Table S4. Performance (BA) of individual structures from SEPPA 3.0 and available peers.

Glycoprotein antigen

No.	PDB id	SEPPA3.0	SEPPA2.0	Pepito	Epitopia	Discotope2.0	CBTOPE	Bepipred2.0
**1**	3JCX_A	0.579	0.519	0.715	0.596	0.732	0.489	0.541
**2**	5B3J_A	0.662	0.529	0.552	0.644	0.589	0.424	0.639
**3**	5B3J_D	0.588	0.462	0.508	0.632	0.512	0.426	0.540
**4**	5B8C_C	0.599	0.583	0.560	0.425	0.567	0.505	0.593
**5**	5FUU_C	0.530	0.565	0.448	0.707	0.563	0.494	0.600
**6**	5FUU_F	0.586	0.610	0.522	0.617	0.636	0.469	0.638
**7**	5FV1_V	0.590	0.592	0.609	0.517	0.699	0.664	0.569
**8**	5FV2_X	0.691	0.610	0.561	0.526	0.685	0.590	0.527
**9**	5FYL_G	0.776	0.605	0.648	0.693	0.693	0.472	0.605
**10**	5FYL_B	0.592	0.474	0.586	0.566	0.565	0.463	0.612
**11**	5GGR_Z	0.637	0.868	0.646	0.579	0.761	0.554	0.761
**12**	5GGS_Z	0.637	0.595	0.558	0.416	0.525	0.463	0.561
**13**	5GGV_Y	0.503	0.574	0.558	0.396	0.547	0.472	0.576
**14**	5GJS_A	0.656	0.637	0.551	0.640	0.601	0.588	0.545
**15**	5GJS_B	0.721	0.475	0.527	0.508	0.448	0.431	0.596
**16**	5GJT_A	0.714	0.644	0.454	0.639	0.505	0.634	0.594
**17**	5GJT_B	0.623	0.456	0.532	0.479	0.437	0.447	0.577
**18**	5GRJ_A	0.608	0.465	0.448	0.575	0.519	0.576	0.545
**19**	5GS0_A	0.584	0.409	0.491	0.669	0.489	0.662	0.572
**20**	5GZN_A	0.502	0.602	0.608	0.573	0.643	0.587	0.507
**21**	5H37_A	0.808	0.653	0.507	0.666	0.502	0.694	0.635
**22**	5HDQ_A	0.442	0.781	0.556	0.695	0.684	0.461	0.413
**23**	5HHX_A	0.551	0.561	0.699	0.449	0.763	0.545	0.583
**24**	5HI3_B	0.888	0.728	0.660	0.497	0.753	0.547	0.568
**25**	5HI4_A	0.588	0.584	0.695	0.470	0.714	0.545	0.593
**26**	5HI5_A	0.592	0.644	0.802	0.500	0.739	0.259	0.782
**27**	5HJ3_D	0.612	0.761	0.507	0.560	0.717	0.875	0.737
**28**	5I6X_A	0.416	0.489	0.484	0.822	0.568	0.661	0.868
**29**	5I6Z_A	0.480	0.488	0.482	0.821	0.568	0.739	0.867
**30**	5I74_A	0.458	0.528	0.485	0.823	0.585	0.711	0.869
**31**	5I8H_C	0.798	0.508	0.641	0.675	0.682	0.489	0.614
**32**	5I8H_B	0.620	0.777	0.435	0.501	0.623	0.500	0.646
**33**	5I9Q_G	0.754	0.588	0.611	0.532	0.695	0.506	0.637
**34**	5IES_C	0.766	0.746	0.554	0.644	0.554	0.592	0.637
**35**	5IF0_G	0.872	0.692	0.600	0.649	0.593	0.610	0.645
**36**	5IGX_G	0.757	0.615	0.580	0.687	0.736	0.517	0.632
**37**	5J3D_K	0.668	0.714	0.609	0.567	0.610	0.724	0.547
**38**	5J3H_E	0.835	0.406	0.494	0.602	0.500	0.784	0.555
**39**	5J56_A	0.687	0.850	0.501	0.668	0.499	0.341	0.579
**40**	5J57_A	0.528	0.740	0.603	0.661	0.601	0.604	0.683
**41**	5JQ6_A	0.478	0.648	0.623	0.610	0.693	0.527	0.548

**42**	5JS9_C	0.807	0.391	0.527	0.682	0.509	0.377	0.649
**43**	5JS9_D	0.792	0.530	0.778	0.547	0.735	0.482	0.564
**44**	5JSA_D	0.804	0.467	0.792	0.554	0.733	0.483	0.542
**45**	5JW3_A	0.807	0.800	0.553	0.647	0.528	0.436	0.499
**46**	5JW3_B	0.628	0.572	0.416	0.522	0.605	0.490	0.505
**47**	5JW4_A	0.798	0.623	0.511	0.634	0.728	0.583	0.507
**48**	5JXE_B	0.545	0.654	0.605	0.601	0.601	0.476	0.593
**49**	5JYL_A	0.838	0.741	0.637	0.601	0.654	0.425	0.624
**50**	5JYM_A	0.566	0.732	0.551	0.557	0.535	0.553	0.539
**51**	5JZ7_B	0.653	0.631	0.506	0.580	0.545	0.420	0.511
**52**	5K59_A	0.653	0.684	0.688	0.645	0.637	0.466	0.570
**53**	5K9K_I	0.491	0.547	0.460	0.638	0.470	0.415	0.549
**54**	5K9O_I	0.633	0.574	0.445	0.637	0.458	0.483	0.571
**55**	5K9Q_F	0.772	0.548	0.540	0.521	0.605	0.312	0.572
**56**	5KAN_C	0.611	0.477	0.453	0.635	0.447	0.672	0.582
**57**	5KAN_D	0.755	0.538	0.576	0.518	0.606	0.315	0.581
**58**	5KAQ_B	0.784	0.434	0.517	0.588	0.504	0.356	0.624
**59**	5KEL_B	0.725	0.472	0.498	0.584	0.670	0.874	0.759
**60**	5KEL_A	0.558	0.481	0.636	0.622	0.627	0.478	0.620
**61**	5KEM_A_1	0.675	0.700	0.563	0.540	0.558	0.480	0.594
**62**	5KEM_A_2	0.492	0.602	0.547	0.636	0.556	0.434	0.564
**63**	5KEN_E	0.644	0.478	0.653	0.633	0.598	0.472	0.503
**64**	5KEN_B	0.544	0.500	0.597	0.539	0.706	0.845	0.687
**65**	5KJR_G	0.565	0.575	0.521	0.512	0.580	0.386	0.623
**66**	5KOV_A	0.446	0.792	0.498	0.645	0.470	0.519	0.612
**67**	5KTE_A	0.500	0.480	0.495	0.799	0.500	0.664	0.686
**68**	5KW9_A	0.570	0.558	0.606	0.655	0.783	0.561	0.570
**69**	5KZC_A	0.744	0.667	0.559	0.632	0.559	0.519	0.650
**70**	5L0Q_A	0.653	0.539	0.586	0.581	0.531	0.456	0.566
**71**	5L6Y_C	0.648	0.659	0.516	0.565	0.494	0.465	0.521
**72**	5M94_A	0.755	0.637	0.485	0.766	0.478	0.493	0.675
**73**	5MVZ_U	0.567	0.468	0.515	0.596	0.461	0.744	0.552
**74**	5SV3_B	0.715	0.756	0.539	0.619	0.509	0.547	0.608
**75**	5SX4_N	0.803	0.584	0.478	0.596	0.481	0.716	0.610
**76**	5SX5_N	0.775	0.509	0.479	0.602	0.482	0.720	0.630
**77**	5T33_G	0.706	0.773	0.652	0.668	0.822	0.419	0.690
**78**	5T3S_G	0.803	0.408	0.464	0.651	0.452	0.426	0.583
**79**	5T3X_G_1	0.842	0.509	0.598	0.678	0.659	0.413	0.565
**80**	5T3X_G_2	0.595	0.522	0.606	0.617	0.607	0.521	0.505
**81**	5T3Z_G	0.532	0.498	0.613	0.528	0.630	0.489	0.521
**82**	5TE4_G	0.825	0.639	0.587	0.616	0.703	0.387	0.559
**83**	5TE6_G	0.911	0.814	0.620	0.639	0.709	0.522	0.561
**84**	5TE7_G	0.868	0.693	0.576	0.619	0.636	0.375	0.542
**85**	5TH9_A	0.574	0.825	0.601	0.642	0.620	0.368	0.604

**86**	5THR_C	0.623	0.548	0.655	0.624	0.567	0.447	0.498
**87**	5THR_D	0.806	0.713	0.529	0.609	0.444	0.694	0.543
**88**	5TLJ_X_1	0.526	0.767	0.563	0.581	0.531	0.698	0.555
**89**	5TLJ_X_2	0.472	0.630	0.519	0.567	0.509	0.484	0.562
**90**	5TLK_X_1	0.924	0.788	0.531	0.664	0.531	0.703	0.562
**91**	5TLK_X_2	0.731	0.606	0.509	0.542	0.509	0.491	0.554
**92**	5TOJ_A	0.572	0.610	0.474	0.604	0.534	0.545	0.581
**93**	5TOK_B	0.554	0.606	0.524	0.604	0.467	0.553	0.595
**94**	5TPN_A	0.430	0.387	0.486	0.628	0.462	0.462	0.613
**95**	5TPW_A	0.770	0.414	0.506	0.639	0.484	0.441	0.594
**96**	5TQ0_A	0.836	0.543	0.480	0.624	0.480	0.433	0.565
**97**	5TQ2_A	0.772	0.550	0.512	0.599	0.483	0.446	0.608
**98**	5TQQ_A	0.434	0.645	0.566	0.680	0.552	0.479	0.747
**99**	5TR1_A	0.418	0.612	0.572	0.676	0.572	0.481	0.771
**100**	5TZ2_C	0.632	0.569	0.484	0.624	0.473	0.494	0.546
**101**	5TZT_C	0.589	0.712	0.485	0.570	0.490	0.440	0.532
**102**	5TZU_C	0.443	0.602	0.484	0.594	0.484	0.448	0.547
**103**	5U1F_A	0.916	0.839	0.788	0.671	0.776	0.465	0.583
**104**	5UGY_A	0.751	0.599	0.649	0.641	0.737	0.477	0.489
**105**	5UQY_E	0.571	0.545	0.541	0.563	0.469	0.508	0.588
**106**	5WT9_G	0.817	0.930	0.738	0.398	0.765	0.559	0.790
	Average [a]	0.656	0.604	0.561	0.605	0.587	0.523	0.599

Non-glycoprotein antigen

**No.**	PDB id	SEPPA3.0	SEPPA2.0	Pepito	Epitopia	Discotope2.0	CBTOPE	Bepipred2.0
**107**	5FUO_A	0.524	0.437	0.526	0.645	0.497	0.629	0.651
**108**	5GS1_D	0.683	0.372	0.522	0.408	0.505	0.553	0.595
**109**	5GZO_A	0.616	0.422	0.488	0.570	0.503	0.612	0.537
**110**	5H35_C	0.487	0.752	0.494	0.617	0.500	0.485	0.586
**111**	5HGG_B	0.610	0.617	0.467	0.446	0.478	0.539	0.618
**112**	5HHV_A	0.707	0.580	0.653	0.547	0.720	0.553	0.580
**113**	5IHL_A	0.663	0.679	0.500	0.577	0.503	0.488	0.564
**114**	5JHL_A	0.862	0.855	0.488	0.596	0.517	0.777	0.784
**115**	5KVD_E	0.619	0.627	0.483	0.615	0.470	0.515	0.517
**116**	5KVE_E	0.549	0.651	0.491	0.575	0.514	0.485	0.546
**117**	5KVF_E	0.596	0.710	0.499	0.402	0.509	0.515	0.527
**118**	5KVG_E	0.556	0.529	0.554	0.600	0.572	0.493	0.626
**119**	5LQB_A	0.558	0.522	0.607	0.627	0.607	0.520	0.679
**120**	5MES_A	0.510	0.576	0.536	0.627	0.514	0.550	0.594
**121**	5MEV_A	0.572	0.573	0.540	0.670	0.516	0.525	0.608
**122**	5MI0_A	0.486	0.486	0.599	0.579	0.771	0.691	0.412
**123**	5SY8_O	0.723	0.644	0.466	0.573	0.478	0.432	0.528
**124**	5T29_O	0.752	0.639	0.477	0.459	0.508	0.438	0.471
**125**	5T5B_E	0.609	0.645	0.467	0.508	0.519	0.411	0.451

**126**	5T5F_A	0.558	0.576	0.813	0.652	0.952	0.611	0.591
**127**	5T85_G	0.642	0.685	0.470	0.497	0.485	0.456	0.474
**128**	5TFW_O	0.600	0.663	0.471	0.597	0.514	0.415	0.461
**129**	5TIH_A	0.755	0.639	0.535	0.648	0.535	0.525	0.553
**130**	5WTH_B	0.691	0.768	0.582	0.609	0.582	0.510	0.587
	Average [b]	0.650	0.606	0.555	0.598	0.580	0.524	0.593

[a] Averaged BA value of 106 glycoprotein antigens.

[b] Averaged BA value of 130 general protein antigens.
